# Application of the symbolic regression program AI-Feynman to psychology

**DOI:** 10.3389/frai.2023.1039438

**Published:** 2023-01-27

**Authors:** Masato Miyazaki, Ken-Ichi Ishikawa, Ken'ichiro Nakashima, Hiroshi Shimizu, Taiki Takahashi, Nobuyuki Takahashi

**Affiliations:** ^1^Department of Behavioral Science, Graduate School of Letters, Hokkaido University, Sapporo, Hokkaido, Japan; ^2^Graduate School of Advanced Science and Engineering, Hiroshima University, Higashi-Hiroshima, Hiroshima, Japan; ^3^Department of Psychology, Graduate School of Humanities and Social Sciences, Hiroshima University, Higashi-Hiroshima, Hiroshima, Japan; ^4^Faculty of Sociology, Kwansei Gakuin University, Nishinomiya, Hyogo, Japan

**Keywords:** time preference, symbolic regression, AI-Feynman, hyperbolic discounting model, artificial intelligence

## Abstract

The discovery of hidden laws in data is the core challenge in many fields, from the natural sciences to the social sciences. However, this task has historically relied on human intuition and experience in many areas, including psychology. Therefore, discovering laws using artificial intelligence (AI) has two significant advantages. First, it makes it possible to detect laws that humans cannot discover. Second, it will help construct more accurate theories. An AI called AI-Feynman was released in a very different field, and it performed impressively. Although AI-Feynman was initially designed to discover laws in physics, it can also work well in psychology. This research aims to examine whether AI-Feynman can be a new data analysis method for inter-temporal choice experiments by testing whether it can discover the hyperbolic discount model as a discount function. An inter-temporal choice experiment was conducted to accomplish these objectives, and the data were input into AI-Feynman. As a result, seven discount function candidates were proposed by AI-Feynman. One candidate was the hyperbolic discount model, which is currently considered the most accurate. The three functions of the root-mean-squared errors were superior to the hyperbolic discount model. Moreover, one of the three candidates was more “hyperbolic” than the standard hyperbolic discount function. These results indicate two things. One is that AI-Feynman can be a new data analysis method for inter-temporal choice experiments. The other is that AI-Feynman can discover discount functions that humans cannot find.

## 1. Introduction

This study uses artificial intelligence that can automatically discover the laws of physics to discover the law of time preference and share its review. Here, the laws are defined as functions that match the data obtained from humans. This paper aims to inform psychologists that AI is a powerful tool to discover laws and that it is simple, easy, and free to use.^1^ To demonstrate the usefulness and possibility of AI in the field of psychology, we will examine a tool named AI-Feynman (Udrescu and Tegmark, 2020; Udrescu et al., 2020), which is a neural-network-based symbolic regression fitting tool developed by physicists, to discover the lows or tendencies from psychological phenomena. As an example, we apply AI-Feynman to explore the lows of time preference and discounting phenomenon^2^ and explain how it works to extract lows from the experimental data. We conduct a single experiment on time preference as an example. Although we only have one experiment, we find seven candidates for the discount function using AI-Feynman, and two of them provide intriguing models and others include known functions. This would provide an effectiveness of AI-Feynman, though the novelty of the two models is limited due to the small sized sample. An application of AI technology (convolutional neural network) to education has been reported in Liu et al. (2022), where the MOOC forum discussions of an E-learning system are analyzed to explore the relationships among emotional and cognitive engagements, and learning achievement.

Section 2 explains the necessity of discovering the laws of time preference. Section 3 introduces AI that can automatically discover physical laws and shows that AI can work well in psychology. In Section 4, AI is used to reveal the discount function from experimental data of inter-temporal choice, and it is shown that the results are consistent with previous findings. Finally, Section 5 discusses our conclusions, limitations, and future directions.

The discovery of laws in psychology is important because it increases the reproducibility of findings and helps develop psychology as a science. There are two methods for discovering laws in psychology. One is the deductive method, which derives laws from the existing theories and axioms. The other is the inductive method, which searches for laws that well explain experimental facts.

An example of the inductive method is the discovery of the laws of time preference in inter-temporal choices. Inter-temporal choice is defined as decisions involving trade-offs between costs and benefits that occur at different times (Frederick et al., 2002). It is also known that people prefer receiving a reward earlier time rather than later (Koopmans, 1960). This phenomenon is known as “time preference” in economics and “delay discounting” in psychology. For example, if people are given two options: (A) get $10 now and (B) get $10 at the end of the month, most people choose (A) even though the amount of money is equal between the two options.

Since such choices can affect economic prosperity on a national scale (Smith, 1976), they have been studied since early in the history of economics (Rae, 1905). However, until 1936, too many theories explained inter-temporal choice, and economists did not understand it systematically. Sub-sequently, the Exponential Discounting Model (EDM) was proposed by Samuelson (1937). His theory significantly advanced understanding, leading to its rapid acceptance because it explained inter-temporal choice with only one concept, “discounting”. Discounting is captured mathematically in the discount function λ(*t*), as shown in Equation (1):


(1) $$\begin{array}{l} U\left(A,t\right)=A\lambda \left(t\right) \end{array}$$

where *t* is the time, *A* is the amount of goods, and *U* is the utility function. The utility function converts the value of receiving *A* at time *t* to utility. λ(*t*) takes values ranging from zero to one. The EDM considers λ(*t*) = *e*^−κ*t*^, where κ is a positive real parameter that determines discount level. In this case, the utility function is expressed by Equation (2).


(2) $$\begin{array}{l} U\left(A,t|\kappa \right)=Ae^{-\kappa t} \end{array}$$

EDM was deduced from the old economic axiom about human decision-making that assumes people are rational^3^ [For details and proof, see Appendix, Section 6.1 A2 of Musau (2009)]. Because of this, EDM views a present bias as an anomaly. The present bias is the reversal of preference over time. People often experience doing a task that they are sure to do tomorrow but put it off. This example typifies present bias because their preference for “doing the job” to “putting it off” is reversed over time.

On the other hand, psychologists inductively discovered the hyperbolic discounting model (HDM) through experiments on humans and pigeons (Green and Myerson, 1996). The HDM considers that λ(*t*) = 1/(1+κ*t*). In this case, the utility function is expressed as Equation (3).


(3) $$\begin{array}{l} U\left(A,t|\kappa \right)=A\frac{1}{1+\kappa t} \end{array}$$

**Figure 1:**
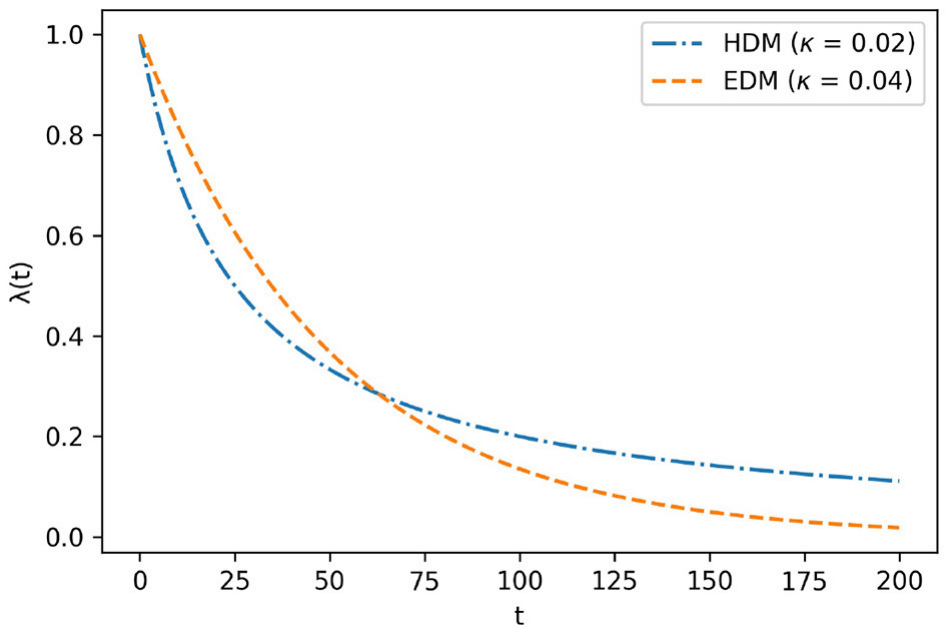
Graph of EDM and HDM.

The graphs of EDM and HDM are shown in Figure 1. Subsequent empirical research revealed that the HDM is more suitable for λ(*t*) than EDM because the former can explain present bias, whereas EDM cannot. The present bias occurs when λ(*t*) is the HDM instead of the EDM. Since people often put off something they would do,^4^ in daily life, HDMs can capture the essence of time preference more than EDMs.

As the example above shows, inductive law discovery is essential because it can discover new theories and axioms founded on real-world phenomena. However, since inductive law discovery has relied on human intuition and experience in many fields, researchers have often overlooked some laws. In particular, when true laws are expressed via complex mathematical formulae, researchers who do not use mathematics as the primary tool in the theoretical analysis might not discover them.

In a very different field, an AI called AI-Feynman, which can discover physical laws, was released, and it achieved impressively high performance (Udrescu and Tegmark, 2020; Udrescu et al., 2020). Although AI-Feynman was initially designed to discover laws in physics, it can also work well in psychology. This study demonstrated that the law of time preference can be found from experimental data with AI-Feynman and can help researchers who do not have high mathematical skills.

## 2. Materials and methods

### 2.1. AI-Feynman

In this section, the AI-Feynman machinery is briefly explained. For more details, please refer to the following references (Udrescu and Tegmark, 2020; Udrescu et al., 2020; Miyazaki, 2021).

#### 2.1.1. Symbolic regression and fitting

The discovery of laws is sometimes referred to as a symbolic regression. Symbolic regression discovers hidden laws as a formula, namely a combination of “symbols”. Strictly speaking, given *n* independent variables {*x*_1_, *x*_2_, …, *x*_*n*_} and one dependent variable *y*, symbolic regression is the process of finding the formula of function *f*, where *y* = *f*(*x*_1_, *x*_2_, …, *x*_*n*_) by searching the formula space (Udrescu and Tegmark, 2020). In contrast, adjusting the arbitrary constants in a particular function to fit the given data is called “fitting.” Fitting can be distinguished from symbolic regression.

For symbolic regression, it is necessary to find *f* as a combination of symbols that maximizes the fit to the data. This process is time-consuming, as the number of combinations increases in the order of *s*^*l*^, where *l* is the length of *f* as a symbolic combination, and *s* is the number of possible symbols for *f*. This trend implies that the formula space to be searched is generally vast. However, AI-Feynman uses a neural network to detect the symmetry and separability of *f*. As a result, it recursively reduces the formula space. Therefore, it is possible to avoid searching for a vast formula space with relative ease.

Until AI-Feynman was released in April 2020, the Eureqa software (Schmidt and Lipson, 2009; Praksova, 2011) was the best symbolic regression program. Both programs were tested to regress 100 laws symbolically *Feynman's Lecture on Physics* (Feynman, 1963a,b; Feynman et al., 1963). As a result, Eureqa symbolically regressed only 71 laws, while AI-Feynman achieved symbolic regression of 100 laws. There are two versions of AI-Feynman: the unbranded AI-Feynman, released in April 2020, and the AI-Feynman 2.0 released in September 2020. In this research, we have attempted to explain AI-Feynman 2.0, because it recorded higher performance. Therefore, when we use the word “AI-Feynman” in the following text, it means AI-Feynman 2.0. The source code of AI-Feynman 2.0 is at https://github.com/dcshapiro/AI-Feynman.

#### 2.1.2. Outline of algorithm

As mentioned in the previous section, symbolic regression takes a long time because the formula space increases in the order of *s*^*l*^. However, functions appearing in many scientific fields, including physics and psychology, often have the following three properties introduced in Udrescu and Tegmark (2020). These properties enable us to shorten the time required for symbolic regression.

1. Smoothness^5^: A formula is continuous and analytic in the domain of the given data.(e.g., The formula for kinetic energy and distance between two points is analytic within the domain of definition).
2. Symmetry: A formula exhibits either translational, rotational, or scaling symmetry for some variables.(e.g., The formula of distance between two points $d=\sqrt{{\left(x_{1}-x_{2}\right)}^{2}+{\left(y_{1}-y_{2}\right)}^{2}}$ has the symmetry of being invariant to the translational transformation *x*_1_→*x*_1_+*a, x*_2_→*x*_2_+*a* with any constant *a*).
3. Separability: A formula can be written as the sum or product of two parts that have no variables in common.(e.g., The formula for kinetic energy $\frac{mv^{2}}{2}$ can be written as the product of the term $\frac{m}{2}$ about *m* and the term *v*^2^ about *v*).

The formula space to search can be reduced using the properties of *f*. Using property (i) enables us to approximate *f* with a feed-forward neural network^6^ (FNN), whose activation function is analytical.^7^ Using the FNN in (i) can confirm the presence or absence of (ii). If we confirm that *f* has symmetry, the number of independent variables can be reduced by one. The presence or absence of (iii) can also be confirmed using the FNN. If it is confirmed that *f* has separability, it can be divided into two simpler independent functions.

**Figure 2:**
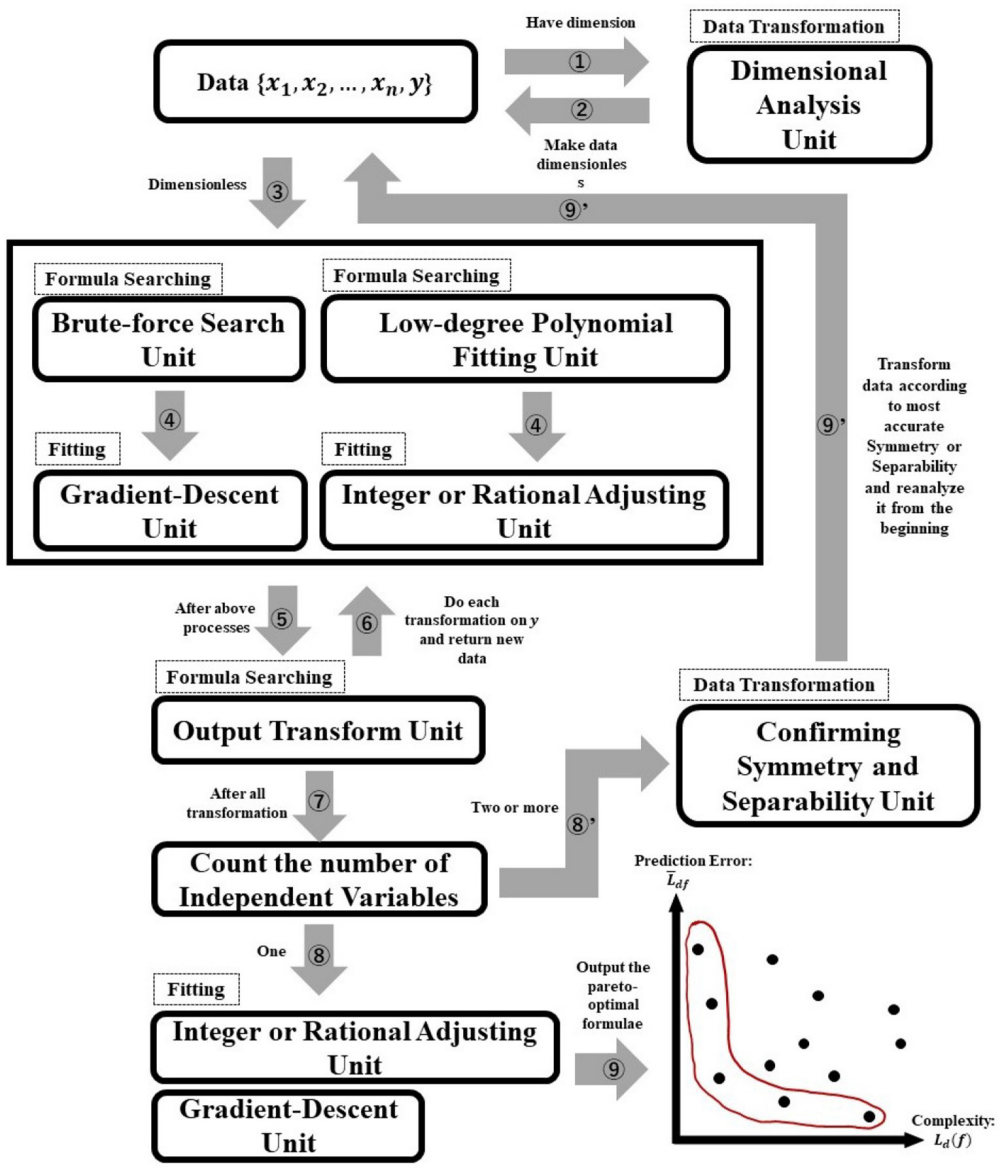
Overview of AI-Feynman's algorithm.

An overview of AI-Feynman's algorithm is presented in Figure 2. As can be seen in Figure 2, the AI-Feynman algorithm consists of seven units (dimensional analysis, brute-force search, gradient-descent, low-degree polynomial fitting, integer or rational adjusting, output transform, confirming symmetry and separability units). These units help AI-Feynman to shorten the time of symbolic regression. The dashed squares indicate the role of each unit (data transformation, formula searching, or fitting), and the numbers attached to the arrows indicate the order of the process (the process starts with the arrow that has the number 1). The essential thing in Figure 2 is that AI-Feynman can discover the laws behind data regardless of whether they are laws in physics or psychology because the data you input into AI-Feynman are only combinations of numbers (and units).

Because of space limitations, the details of AI-Feynman are not presented here. Interested readers can refer to a brief review of AI-Feynman (Miyazaki, 2021).

### 2.2. Experiment

The data of the discount function λ(*t*) must be collected by changing the value of *t*, namely $\left\{t^{i},\lambda {\left(t\right)}^{i},i=1,\ldots ,N_{d}\right\}$.

The data {(*t*^*i*^, λ(*t*)^*i*^)} can be collected by asking “At least how much money would you like to receive now compared to receiving *y* [yen] after *t* days later?” varying the value of *t*. If the answer is *x*[yen], Equation (4) is provided.


(4) $$\begin{array}{l} \begin{array}{c} y\lambda \left(t\right)=x\lambda \left(0\right)\\ \lambda \left(t\right)=\frac{x}{y}\;\left(∵\lambda \left(0\right)=1\right) \end{array} \end{array}$$

**Figure 3:**
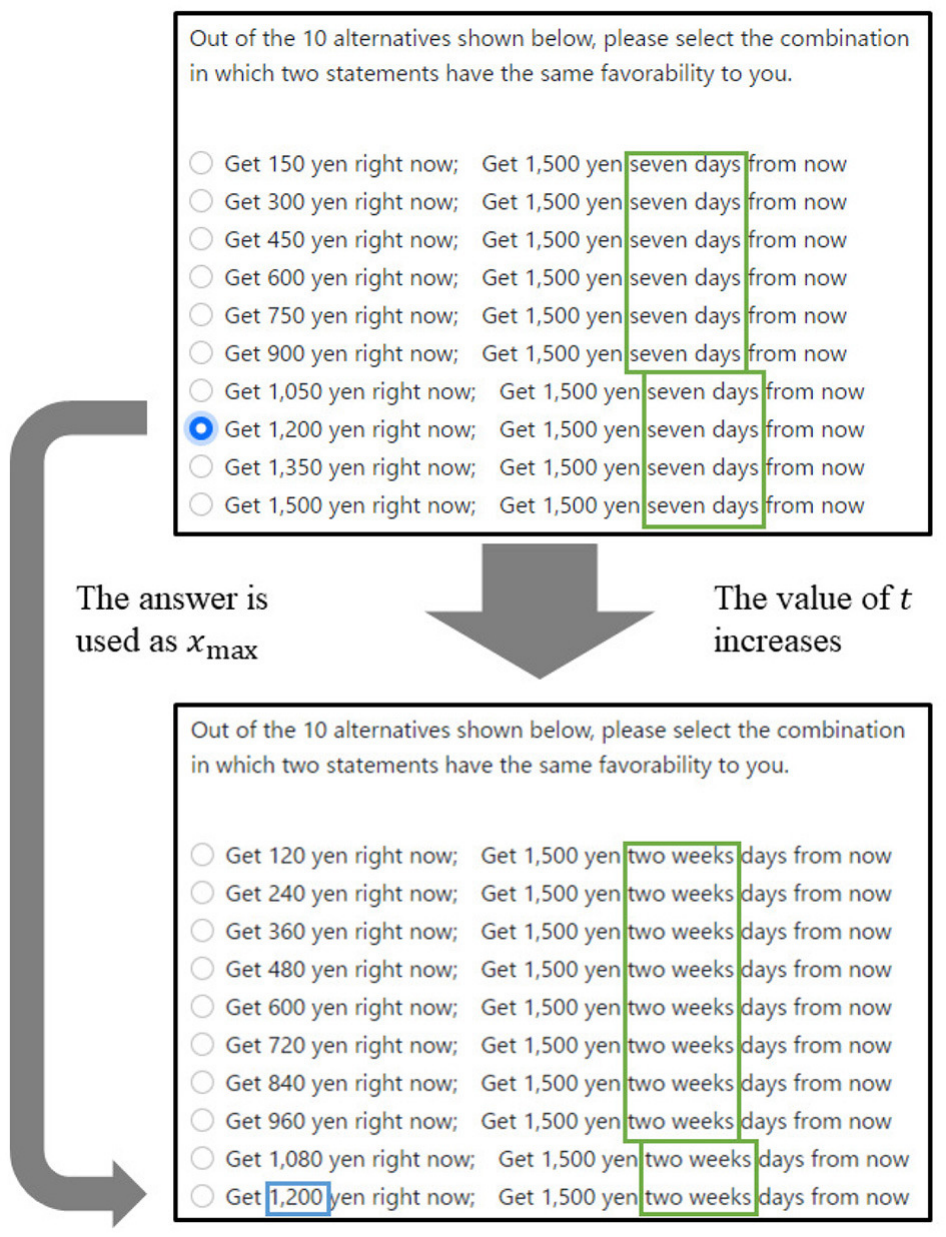
Example of questionnaire's first two steps.

However, in practice, it is difficult for participants to determine accurately the minimum value of money. Therefore, in this study, data were collected using the 10-choice question shown in Figure 3. This question type was repeated several times by changing *y* and *t*. Each of the values of *y*[yen] and *t*[days] used in experiment varies as 1,500, 4,000, 10,000, 25,000, 300,000, 500,000, and 1,000,000 and 7, 14, 21, 30, 60, 90, 120, 150, 180, 210, 240, 270, 300, 330, and 360 respectively.^8^ Because the experiment was conducted for all combinations of *y* and *t*, the participants answered 105 times in total.

The value of *t* increased from seven to 360. Additionally, because the discount function is a decreasing function, the answer at time *t*_1_ can be regarded as the maximum value of the answer *x*_max_[yen] at the next time *t*_2_. For example, it was unlikely that a participant who chose 1,200 yen on the upper sheet in Figure 3 would choose more than 1,200 yen on the lower sheet in Figure 3. Therefore, if the answer to the questionnaire described in the top panel of Figure 3 were 1,200 yen, the *x*_max_ of next questionnaire in the bottom panel of Figure 3 would be 1,200 yen. As mentioned earlier, there were 105 questions in total, and the experiment took approximately 20 minutes to complete. The experiment was conducted in a room at Hiroshima University, and the questions were displayed on a computer screen.

## 3. Results

The participant in the current experiment was the first author. Only one subject participated in the experiment because the primary objective of this research was to determine whether AI-Feynman estimated the discount function correctly.

**Figure 4:**
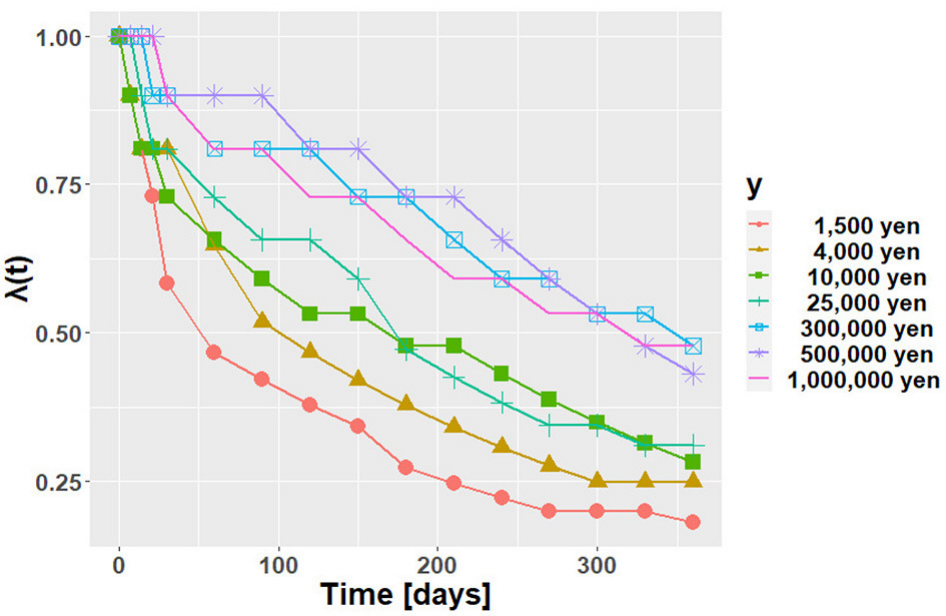
The results of our first author's discount function.

The experimental results are shown in Figure 4. The vertical and horizontal axes represent λ(*t*) and time, *t*, respectively. Each line shows the results for each *y*. From Figure 4, we can observe that the future value is discounted. It can also be seen that the larger the y is, the larger the value of the discount function. This phenomenon is known as the magnitude effect; the value of a discount function is greater for large-magnitude goods (Loewenstein and Prelec, 1992).

Because of the magnitude effect, the data were input for each value of *y* into AI-Feynman. AI-Feynman regressed multiple discount functions. These are shown in Table 1. Let them be *f*_*i, y*_(*t*). The root mean squared error (RMSE) was then calculated for each function defined by Equation (5).


(5) $$\begin{array}{l} \text{RMS}{\text{E}}_{i}\equiv \sqrt{\frac{\underset{y}{\sum }{\left\{f_{i,y}\left(t\right)-\lambda \left(t\right)\right\}}^{2}}{N\left(\text{totalnumberofthequestions}\right)}} \end{array}$$

**Table 1:** Function forms regressed by AI-Feynman.

**Function form**	**Name**	**RMSE**	**Present bias**
$\sqrt{\cos\left(\frac{1}{4}\log\left(\kappa_{1}t+1\right)\right)}$	Model A	6.4 × 10^−2^	Have
$1-\sqrt{\kappa_{1}t}$	Model B	5.6 × 10^−2^	None
$\cos\left(\kappa_{1}t^{\frac{1}{4}}\right)$	Model C	5.4 × 10^−2^	None
$\frac{1}{1+\kappa_{1}t}$	HDM	4.0 × 10^−2^	Have
$\sin\left(\frac{1}{\sqrt{\kappa_{1}t+\kappa_{2}}}\right)+1-\sin\left(\frac{1}{\sqrt{\kappa_{2}}}\right)$	Model D	4.0 × 10^−2^	Have
$\kappa_{3}\cdot e^{-\kappa_{1}t}+\left(1-\kappa_{3}\right)$	Model E	3.7 × 10^−2^	Have
$\kappa_{3}\cdot \frac{1}{1+\kappa_{1}t}+\left(1-\kappa_{3}\right)$	Model F	3.1 × 10^−2^	Have
*e*^κ*t*^ (Reference Only)	EDM	6.3 × 10^−2^	None

**Figure 5:**
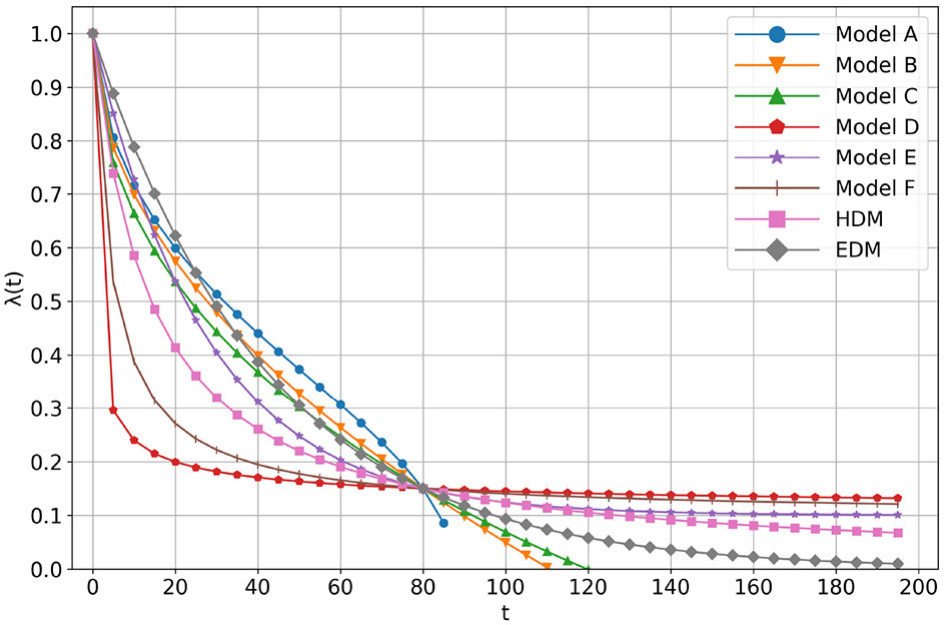
The shape of functions regressed by AI-Feynman.

The calculation results are shown in Table 1 in the presence or absence of the present bias. Note that κ_1_ is a positive constant, κ_2_ is a constant s.t. $\frac{\pi^{2}}{4}\leq \kappa_{2}$, and κ_3_ is a constant s.t. 0 ≤ κ_3_ ≤ 1. Although EDM was not provided by AI-Feynman, it was in Table 1 for reference only. The shapes of the regressed functions are shown in Figure 5. All the coefficients of each model are adjusted so that they cross at (*t*, λ(*t*)) = (80, 0.15).

### 3.1. Discovery of the model which is more “hyperbolic” than HDM

As can be seen in the fifth line of Table 1, the HDM was regressed. As a result, Models D, E, and F have present biases, and their RMSEs are below that of the HDM.^9^ Therefore, we treat Models D, E, and F as candidates superior to HDM. In the following, the properties of Models D, E, and F are described in detail. On the other hand, Model A is inappropriate as λ(*t*) because its RMSE value is inferior to that of HDM. Models B and C are also inappropriate as λ(*t*) because they do not have a present bias.

**Figure 6:**
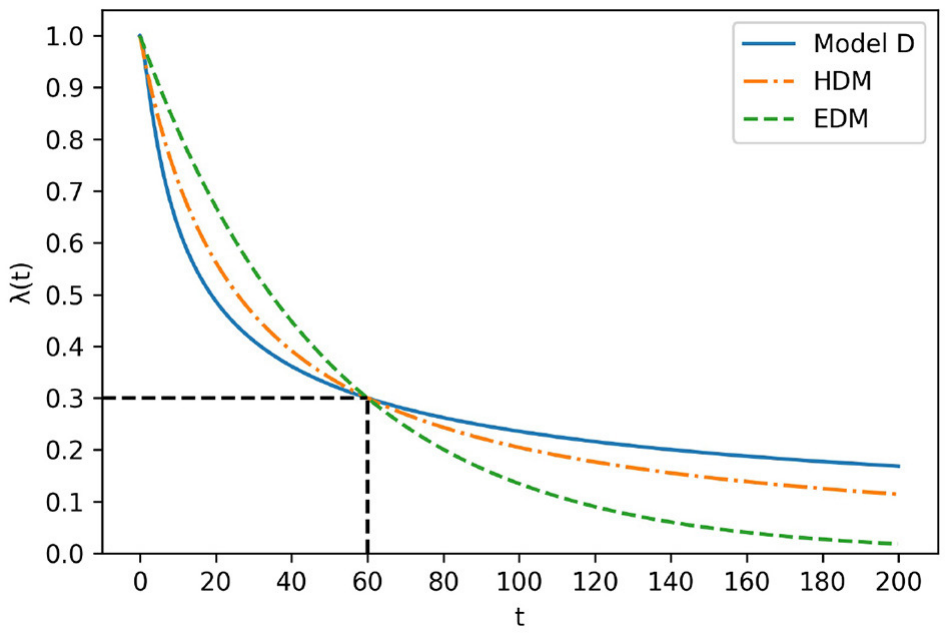
Comparison of model D, HDM, and EDM.

First, Model D is differentiable in the domain of *t*≥0 because $0<\sin\left(\frac{1}{\sqrt{\kappa_{1}t+\kappa_{2}}}\right)\leq \sin\left(\frac{1}{\sqrt{\kappa_{2}}}\right)<1$ from definition $\kappa_{1}>0,\kappa_{2}>\frac{4}{\pi^{2}}$. This implies that Model D satisfies the definition of λ(*t*). Additionally, the value of Model D is closer to $1-\sin\left(\frac{1}{\sqrt{\kappa_{2}}}\right)$ at *t* → ∞. Figure 6 illustrates the shape of Model D with EDM and HDM. All coefficients of each model are adjusted such that they cross at (*t*, λ(*t*)) = (60, 0.3). As shown in Figure 6, model D drops more sharply than the HDM around *t* < 60 and drops more gently around 60 < *t*. This feature indicates that Model D is a more “hyperbolic” model than HDM.

Model E can be understood as a mixed model of EDM and 1. In other words, Model E is the sum of $e^{-\kappa_{1}t}$ with a weight of κ_3_ and one with a weight of 1−κ_3_. Model E can be understood as one that exponentially discounts with probability κ_3_ and does not discount at all with probability 1−κ_3_.

Model F is also understood as a mixed model of HDM and 1. This means that Model F hyperbolically discounts with probability κ_3_ and does not discount at all with probability 1−κ_3_.

## 4. Discussion

The AI-Feynman regressed the HDM. This result indicates that AI-Feynman can be a new data analysis method for inter-temporal choice experiments. AI-Feynman also regressed other intriguing models, such as models E and F. These are mixed models composed of EDM or HDM and a no-discount model. Such models have never been proposed before, suggesting that that AI-Feynman can discover new laws.

### 4.1. Evaluating regression results with domain knowledge

Here, we emphasize that domain knowledge can be used to evaluate the regression results of AI-Feynman. For example, in the previous section, Model C was evaluated as inappropriate as λ(*t*) because it does not have a present bias. However, even if there is a present bias, Model C can be rejected using the domain knowledge of λ(*t*). First, by definition, λ(*t*) is a monotonically decreasing function. However, cos is not a monotonically decreasing function. Therefore, Model C is not appropriate as λ(*t*).

### 4.2. New study procedure

**Figure 7:**
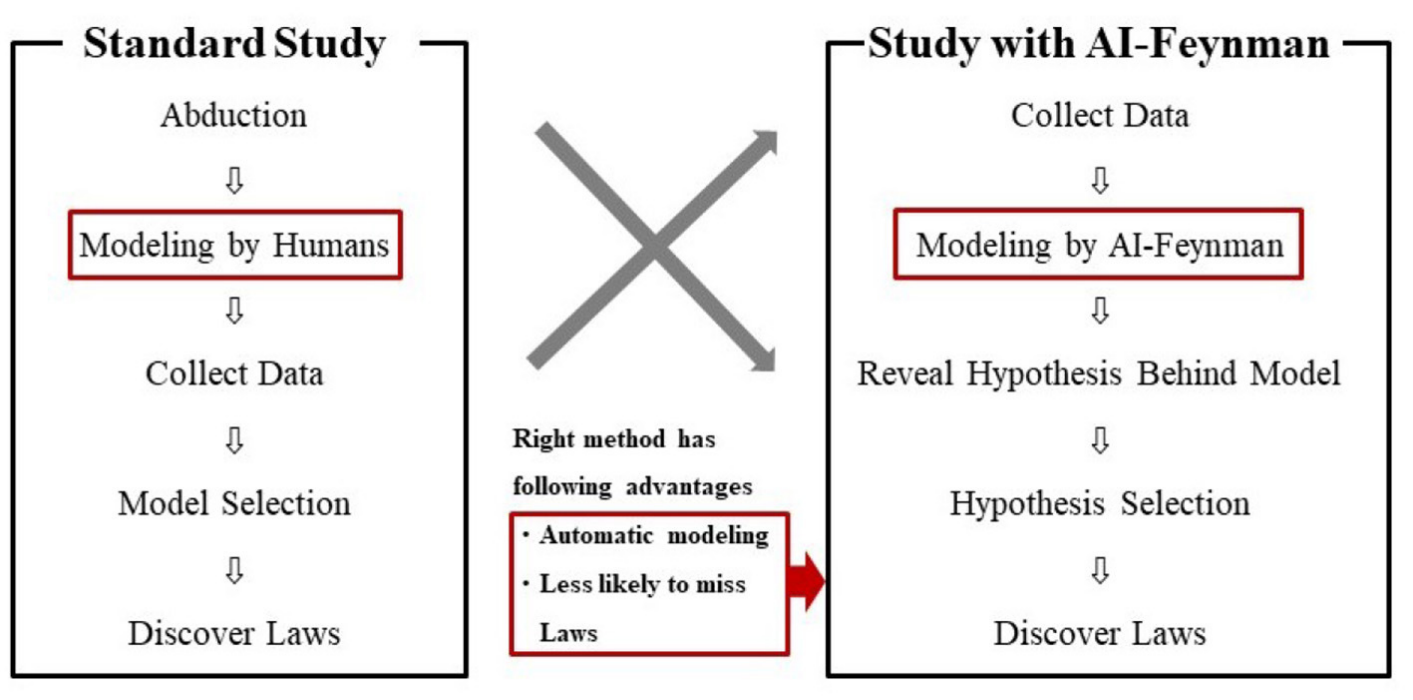
Comparison of standard study and study with AI-Feynman.

In this study, we demonstrated a new study procedure using AI-Feynman. The new procedure was compared with the standard one in Figure 7. As shown on the left side of Figure 7, a standard study was conducted in the following sequence: abduction, modeling by humans, data collection, model selection, and discovery of laws. On the other hand, the study with AI-Feynman, as shown on the right side of Figure 7, contains the following sequence: collecting data, modeling by AI-Feynman, revealing hypotheses behind the model, selecting hypotheses, and discovering laws. A study with AI-Feynman has the advantages of “automatic modeling” and “less likely to miss laws.”

### 4.3. Future studies

The findings obtained from this study were based on data from only one participant. Therefore, the same experiment should be conducted with various participants whose discount functions would vary. Individual differences must be considered when collecting data from more than one person.

When data were collected from more than one person, there were individual differences. The type of analysis used depends on whether individual differences are assumed. If individual differences are not assumed in a specific field, the mean of each participant's data should be input into AI-Feynman. This analysis is equivalent to the fixed-effects model (Hsiao, 2003, pp. 51–52).

On the other hand, if individual differences are assumed, it is desirable to remodel AI-Feynman to discover a model whose arbitrary constants in discount functions differ for each participant. For example, the HDM is $\frac{1}{1+\alpha_{i}t}$, where α_*i*_ represents an arbitrary constant for each participant. Therefore, it is desirable to remodel the AI-Feynman so that $\frac{1}{1+\alpha_{i}t}$ will be regressed.

## Data availability statement

The datasets presented in this study are publicly available. This data can be found here: https://osf.io/qajfz/.

## Ethics statement

Ethical review and approval was not required for the study on human participants in accordance with the local legislation and institutional requirements. Written informed consent for participation was not required for this study in accordance with the national legislation and the institutional requirements. Written informed consent was not obtained from the individual(s) for the publication of any potentially identifiable images or data included in this article.

## Author contributions

MM, K-II, HS, and TT: conceptualization. MM, KN, and HS: methodology. NT: supervisation. All authors contributed to the article and approved the submitted version.

## Footnotes

^1^This work is based on and extended from the first author's under-graduate thesis (Miyazaki, 2021).

^2^We do not provide details of time preference and discounting phenomenon and interested readers are referred to Frederick et al. (2002).

^3^In this context, the word “humans are rational” means that the value of humans' subjective interest rate does not change with time.

^4^“Eating high-calorie meals while dieting” and “spending money you would save” are also examples of present bias.

^5^The quasi-hyperbolic discounting model (Ikeda, 2012, pp. 79–81) does not satisfy this condition and cannot be symbolically regressed.

^6^This is a standard neural network whose signals propagate only from the input to the output.

^7^AI-Feynman's activation function is $\tanh\left(x\right)=\frac{e^{x}-e^{-x}}{e^{x}+e^{-x}}$.

^8^1,000 yen is about $9; checked on July 17, 2021.

^9^The smaller the RMSE, the better the model.
